# Smoking, alcohol, and colon cancer survival are modified by immune biomarkers: a population-representative study

**DOI:** 10.1093/carcin/bgag006

**Published:** 2026-02-03

**Authors:** Yasemin Adali, Maurice B Loughrey, Stephanie Craig, Ronan T Gray, Jacqueline A James, Manuel Salto-Tellez, Philip D Dunne, Helen G Coleman

**Affiliations:** School of Medicine, Dentistry and Biomedical Sciences, Centre for Public Health, Queen’s University Belfast, Belfast BT12 6BA, United Kingdom; Department of Biophysics, Faculty of Medicine, Pamukkale University, Denizli 20160, Türkiye; School of Medicine, Dentistry and Biomedical Sciences, Centre for Public Health, Queen’s University Belfast, Belfast BT12 6BA, United Kingdom; Patrick G Johnston Centre for Cancer Research, Queen’s University, Belfast, Northern Ireland BT9 7AE, United Kingdom; Department of Cellular Pathology, Belfast Health and Social Care Trust, Belfast, Northern Ireland BT12 6BA, United Kingdom; Patrick G Johnston Centre for Cancer Research, Queen’s University, Belfast, Northern Ireland BT9 7AE, United Kingdom; Precision Medicine Centre of Excellence, Queen’s University Belfast, Northern Ireland BT9 7BL, United Kingdom; School of Medicine, Dentistry and Biomedical Sciences, Centre for Public Health, Queen’s University Belfast, Belfast BT12 6BA, United Kingdom; Patrick G Johnston Centre for Cancer Research, Queen’s University, Belfast, Northern Ireland BT9 7AE, United Kingdom; Precision Medicine Centre of Excellence, Queen’s University Belfast, Northern Ireland BT9 7BL, United Kingdom; Northern Ireland Biobank, Queen’s University Belfast, Northern Ireland BT9 7BL, United Kingdom; Regional Molecular Diagnostic Service, Belfast Health and Social Care Trust, Belfast, Northern Ireland BT12 6BA, United Kingdom; Patrick G Johnston Centre for Cancer Research, Queen’s University, Belfast, Northern Ireland BT9 7AE, United Kingdom; Precision Medicine Centre of Excellence, Queen’s University Belfast, Northern Ireland BT9 7BL, United Kingdom; Regional Molecular Diagnostic Service, Belfast Health and Social Care Trust, Belfast, Northern Ireland BT12 6BA, United Kingdom; Integrated Pathology Unit, Institute of Cancer Research, London SW7 3RP, United Kingdom; Patrick G Johnston Centre for Cancer Research, Queen’s University, Belfast, Northern Ireland BT9 7AE, United Kingdom; CRUK Scotland Institute, Glasgow G61 1BD, United Kingdom; School of Medicine, Dentistry and Biomedical Sciences, Centre for Public Health, Queen’s University Belfast, Belfast BT12 6BA, United Kingdom; Patrick G Johnston Centre for Cancer Research, Queen’s University, Belfast, Northern Ireland BT9 7AE, United Kingdom

**Keywords:** colon cancer, immune biomarkers, smoking, alcohol, survival, epidemiology

## Abstract

Lifestyle factors such as smoking and alcohol may influence colon cancer (CC) survival, but it is unclear whether their effects vary by tumour-infiltrating immune biomarkers. This study examined CC-specific survival by smoking and alcohol status, stratified by immune cell density, in a large population-based cohort. The study included 661 individuals who underwent surgery for stage II or III CC between 2004 and 2008 within two Health and Social Care (HSC) Trusts in Northern Ireland. Representative formalin-fixed, paraffin-embedded (FFPE) tumour blocks were retrieved, and immunohistochemistry (IHC) was performed on tissue microarrays constructed from both the central tumour and the invasive margin. Cox proportional hazards models were used to assess CC-specific survival, adjusting for key clinical and demographic confounders. Ever smoking, compared to never smoking, was associated with poorer CC-specific survival among individuals with lower densities of CD3+, CD4 +, and FOXP3 + tumour-infiltrating immune cells. Among those with higher CD8 + cell density in the central tumour, ever smoking was linked to worse outcomes. Similar patterns were seen in the invasive margin, although these were not all statistically significant. No significant associations were observed between alcohol use and survival across any immune biomarker subgroups. Smoking was associated with poorer survival among patients with CC, and this association appears to be modified by the density of tumour-infiltrating immune cells.

## Teaser

Smoking was associated with adverse colon cancer–specific survival, with evidence of effect modification by tumour-infiltrating immune cell density. No significant associations were observed for alcohol consumption. These findings highlight the potential role of the immune microenvironment in shaping lifestyle-related prognostic effects.

## Graphical Abstract

**Graphical Abstract:**
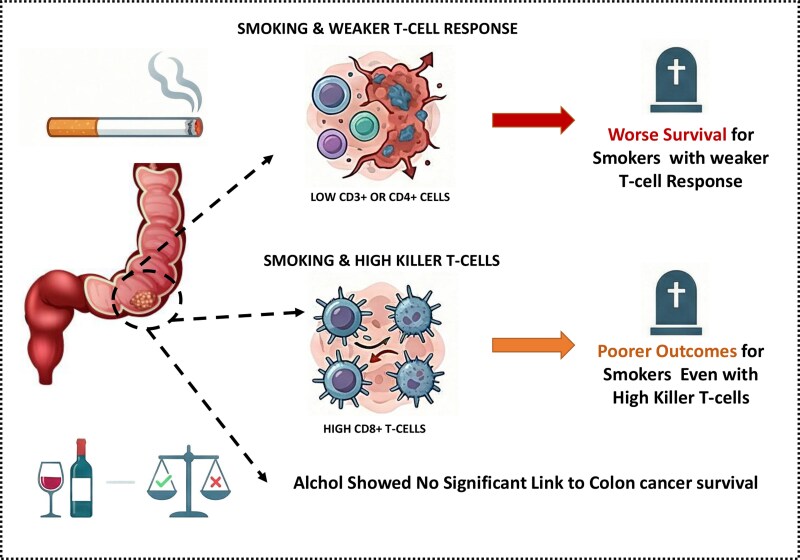

## 1. Introduction

The tumour microenvironment, comprising immune cells, stromal cells, and the extracellular matrix, plays a central role in cancer progression by promoting tumour proliferation, survival, and migration [1]. Over the past two decades, substantial advances have been made in understanding anti-tumour immune responses, leading to new immunotherapeutic approaches in various malignancies [2]. Despite this progress, the mechanisms limiting effective immune responses against cancer remain unclear, and the complex interplay between immune cell subsets continues to be an area of active investigation [2].

A systematic analysis of studies conducted between 1980 and 2012 identified passive antibody therapy and adoptive T-cell transfer as the most effective immunotherapeutic approaches [3]. Among immune components, CD8⁺ cytotoxic T cells and type 1 helper CD4⁺ T cells are considered critical for mounting effective anti-tumour immunity and are generally associated with improved survival outcomes [4]. In contrast, type 2 helper CD4⁺ T cells, which support B-cell-mediated humoral responses, are often linked to poorer prognoses [4]. Tumour-infiltrating lymphocytes (TILs) reflect host immune responses to tumour antigens and are frequently observed in solid tumours, including colorectal cancer (CRC) [5]. Higher densities of TILs, particularly T cells, have been associated with better outcomes in CRC patients [5, 6].

Recently, a comprehensive systematic review and network meta-analysis by Wankhede *et al*. (2024), which included 19 studies and over 13 000 patients with resected CRC, evaluated survival outcomes across four integrated microsatellite instability (MSI) and TILs [6]. The study demonstrated that patients with high TIL levels, regardless of MSI status, had significantly better overall, disease-free, and cancer-specific survival [6]. These findings suggest that TIL density may have stronger prognostic utility than MSI status alone, especially in early-stage CRC, and highlight the need to consider both immune infiltration and molecular subtype in survival analyses [6].

Although inflammation and immune response are increasingly recognized as key factors in tumourigenesis, the impact of modifiable lifestyle factors such as smoking and alcohol consumption on gastrointestinal cancer survival remains incompletely understood [7]. Furthermore, it is unclear whether these lifestyle factors interact with immune cell infiltration to influence prognosis. Investigating the immune contexture of tumours in relation to behavioural exposures may inform novel immunotherapeutic strategies [8].

This study aimed to examine whether the associations between smoking and alcohol consumption and colon cancer (CC)-specific survival differ according to immune biomarker expression within the tumour centre and invasive margin, using data from a population-based cohort study.

## 2. Methods

### 2.1. Study population

The study cohort (EPI700) included 661 patients with stage II or III colon adenocarcinoma diagnosed between 2004 and 2008 across two healthcare trusts in Northern Ireland. Eligible patients were identified through the Northern Ireland Cancer Registry, and surgical resection specimens were retrieved from the Northern Ireland Biobank, as previously described [9–12]. Of 3698 cancer cases initially identified, rectal cancers and stage I or IV cases were excluded. Follow-up for recurrence, cause-specific, and overall mortality was conducted through record linkage with the Northern Ireland Registrar General’s Office until 31 December 2013. Further details on the analytical cohort used in this article are described in the Supplementary file on patient characteristics.

### 2.2. Clinical data collection

Clinical information, including adjuvant chemotherapy, prescription medication use, family history of CRC, and Eastern Cooperative Oncology Group (ECOG) performance status, was obtained from the Clinical Oncology Information System (COIS), a prospective electronic database documenting cancer patient management in Northern Ireland. When data were incomplete or unavailable in COIS, information was supplemented through manual review of hospital case notes. Pathological variables were extracted from full pathology reports. Vital status, date of death, and cause-specific mortality were ascertained through record linkage with the Northern Ireland Registrar General’s Office, with follow-up censored on 31 December 2013. CRC-specific deaths were defined as those with an underlying cause of death coded as ICD-10: C18 (colon), C19 (rectosigmoid junction), C20 (rectum), and/or C26 (other and ill-defined digestive organs).

### 2.3. Smoking and alcohol data collection

Information on lifestyle exposures, including smoking and alcohol use, was extracted from annotated clinical notes recorded at the time of surgical admission and during subsequent treatment. Smoking and alcohol status were initially classified as ‘current’, ‘former’, ‘never’, or ‘unknown’, based on reported exposure at or prior to diagnosis. Due to the limited number of patients identified as former users, smoking and alcohol variables were recategorized as ‘ever’, ‘never’, or ‘unknown’ for the final analysis.

### 2.4. Tissue microarray creation

Following the retrieval of representative FFPE tumour blocks for each CC patient, new sections were cut and stained with haematoxylin and eosin. These slides were reviewed and annotated to guide tissue microarray construction. From each tumour, four representative regions were selected: three from the central tumour and one from the invasive margin. Tissue cores, each 1 mm in diameter, were extracted from the annotated areas and transferred into recipient blocks using a manual tissue arrayer.

### 2.5. Immunohistochemistry and scoring

All immunohistochemistry(IHC) procedures were carried out at the UKAS-accredited Northern Ireland Molecular Pathology Laboratory, which is equipped to process both clinical and research samples [13]. IHC staining for CD3, CD4, CD8, and FOXP3 was performed using automated immunostainers, the Ventana BenchMark XT(Ventana Medical Systems, Oro Valley, AZ, USA) and the Leica BOND-MAX(Leica Biosystems, Wetzlar, Germany). MSI was assessed using five mononucleotide repeat markers (BAT-25, BAT-26, NR-21, NR-24, and MONO-27) following the manufacturer’s protocol of the MSI Analysis System v1.2 kit. MSI testing was conducted at the Northern Ireland Molecular Pathology Laboratory.

### 2.6. QuPath

QuPath is an open-source software platform with a robust user community that supports the development and application of digital pathology tools [14, 15]. Immune cell densities for CD3, CD4, CD8, and FOXP3 were assessed by a pathologist(Maurice B. Loughrey) using QuPath version 0.2.0.m6. The ‘density’ analysis workflow in QuPath was used to evaluate the uniform staining intensity of each biomarker [13, 14]. For each tissue core, the total tissue area (including both tumour and nontumour components) was first identified using the ‘Simple Tissue Detection’ command. Positive cells for each immune marker were then counted using the ‘Fast Cell Count’ tool. Immune cell density was expressed as the number of positive cells per mm², and detection and export processes were automated across all TMA cores using a custom QuPath script [13, 14].

### 2.7. Statistical analysis

All statistical analyses were performed using STATA IC version 16.1 (StataCorp, College Station, TX, USA). Descriptive characteristics of categorical variables were compared using the chi-squared test.

Cox proportional hazards models were used to estimate hazard ratios (HRs) and 95% confidence intervals (CIs) for CRC-specific and overall mortality, comparing alcohol and smoking exposure groups (ever vs never) within strata of immune biomarker expression. For survival analyses, immune biomarker densities (CD3, CD4, CD8, and FOXP3) were dichotomized as low or high based on the median value, separately for the tumour centre and invasive margin.

Multivariable-adjusted Cox models were used to account for potential confounders of colon cancer-specific survival. Covariates included sex; age at diagnosis (<50, 50–<60, 60–<70, 70–<80, >80 years); cancer stage (II or III); tumour differentiation (poorly, well/moderately, or unknown); receipt of adjuvant chemotherapy (yes or no); tumour location (proximal, distal, or unknown); family history of CRC (yes, no, or unknown); ECOG performance status (0–1, 2, 3–4); and microsatellite instability (MSI) status (microsatellite stable [MSS], MSI-high, or unknown). All *P*-values were two-sided, and statistical significance was defined as *P* < 0.05. A sensitivity analysis was conducted by introducing a one-year lag to account for potential reverse causation.

## 3. Results

Median immune cell densities and patient characteristics by CD3, CD4, CD8, and FOXP3 expression at the tumour centre and invasive margin are shown in Supplementary Tables S1–S5. Overall, immune biomarker expression was not associated with age, sex, stage, tumour location, differentiation, chemotherapy, family history, or ECOG status, except that higher CD8 expression was more frequent in right-sided and poorly differentiated tumours, and higher FOXP3 expression was more common in females. As summarized in Table 1, higher expression of all immune biomarkers was linked to improved CRC-specific and overall survival, except for FOXP3 at the tumour centre.

**Table 1:** Colorectal cancer-specific and overall survival by immune biomarker status of stage II and III colon cancer patients.

CRC-specific survival										
		Unadjusted			Adjusted^a^			Adjusted + MSI^b^		
Patients/no. of deaths		HR	(95% CI)	*P*	HR	(95% CI)	*P*	HR	(95% CI)	*P*
**Tumour centre CD3**										
**High vs low**	623/200	0.68	0.51–0.90	**0.01**	0.66	0.49–0.88	**0.01**	0.67	0.50–0.90	**0.01**
**Invasive edge CD3**										
**High vs low**	574/185	0.63	0.47–0.85	**0.01**	0.69	0.51–0.93	**0.02**	0.70	0.52–0.95	**0.02**
**Tumour centre CD4**										
**High vs low**	629/201	0.63	0.47–0.84	**0.01**	0.61	0.46–0.82	**0.01**	0.61	0.46–0.81	**0.01**
**Invasive edge CD4**										
**High vs low**	580/184	0.64	0.47–0.86	**0.01**	0.64	0.47–0.86	**0.01**	0.63	0.46–0.85	**0.01**
**Tumour centre CD8**										
**High vs low**	631/201	0.72	0.54–0.95	**0.02**	0.68	0.51–0.91	**0.01**	0.71	0.53–0.95	**0.03**
**Invasive edge CD8**										
**High vs low**	599/190	0.60	0.45–0.80	**0.01**	0.61	0.45–0.82	**0.01**	0.63	0.46–0.85	**0.01**
**Tumour centre FOXP3**										
**High vs low**	623/200	0.87	0.66–1.16	0.37	0.90	0.68–1.20	0.50	0.89	0.67–1.19	0.46
**Invasive edge FOXP3**										
**High vs low**	558/181	0.67	0.49–0.90	**0.01**	0.69	0.51–0.93	**0.02**	0.69	0.51–0.93	**0.02**

Overall survival										
		Unadjusted			Adjusted^a^			Adjusted + MSI^b^		
Patients/No. of deaths		HR	(95% CI)	*P*	HR	(95% CI)	*P*	HR	(95% CI)	*P*
**Tumour centre CD3**										
**High vs low**	623/291	0.76	0.60–0.96	**0**.**02**	0.73	0.58–0.93	**0**.**01**	0.75	0.59–0.95	**0**.**02**
**Invasive edge CD3**										
**High vs low**	574/272	0.65	0.51–0.83	**0**.**01**	0.70	0.55–0.90	**0**.**01**	0.71	0.55–0.91	**0**.**01**
**Tumour centre CD4**										
**High vs low**	629/292	0.65	0.51–0.82	**0**.**01**	0.62	0.49–0.79	**0**.**01**	0.62	0.49–0.79	**0**.**01**
**Invasive edge CD4**										
**High vs low**	580/269	0.74	0.58–0.95	**0**.**02**	0.74	0.58–0.94	**0**.**02**	0.72	0.56–0.92	**0**.**01**
**Tumour centre CD8**										
**High vs low**	631/292	0.83	0.66–1.05	0.13	0.77	0.60–0.97	**0**.**03**	0.79	0.62–1.01	**0**.**06**
**Invasive edge CD8**										
**High vs low**	599/275	0.66	0.51–0.84	**0**.**00**	0.68	0.54–0.87	**0**.**00**	0.70	0.55–0.90	**0**.**01**
**Tumour centre FOXP3**										
**High vs low**	623/288	0.95	0.75–1.20	0.68	0.94	0.74–1.19	0.66	0.94	0.74–1.20	0.66
**Invasive edge FOXP3**										
**High vs low**	558/262	0.68	0.53–0.86	**0**.**01**	0.67	0.52–0.87	**0**.**01**	0.67	0.52–0.87	**0**.**01**

- Excluded patients with a previous history of CRC, or hereditary-linked CRC from the analysis.

- Unknown tumour centre and maximum immune biomarker status removed.

^a^Adjusted; sex, chemotherapy, age, stage, ECOG status, and tumour grade differentiation.

^b^Adjusted + MSI; sex, chemotherapy, age, stage, ECOG status, tumour grade differentiation, and MSI.

*P*, *P* value.

Bold values indicate statistically significant differences (*P* < 0.05)

### 3.1. Cigarette consumption and survival analysis by immune biomarkers

**Figure 1:**
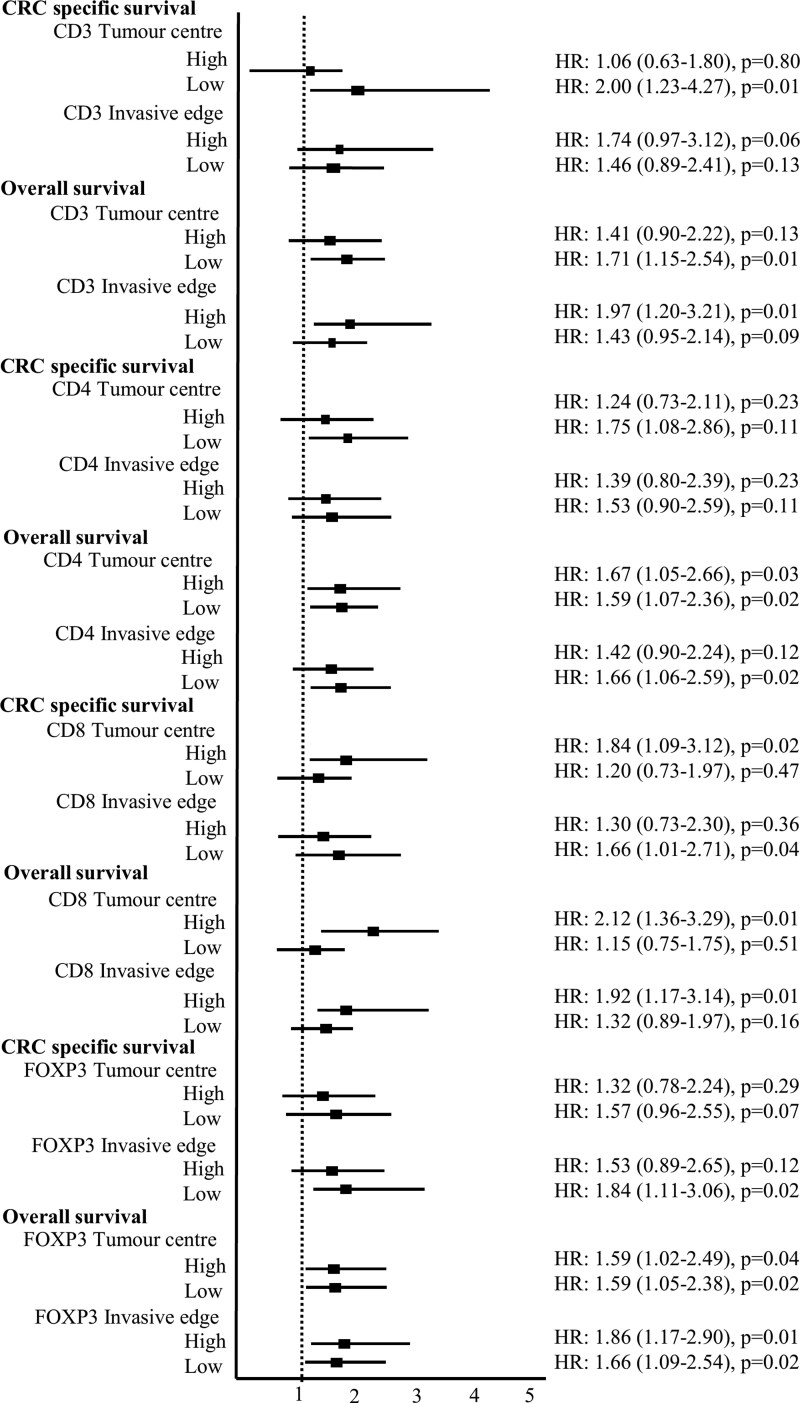
Smoking and colorectal cancer-specific and overall survival by immune biomarker status at tumour centre and invasive edge.

Figure 1 presents the forest plot of the association between ever smoking and CRC-specific and overall survival, stratified by immune biomarker density at the tumour centre and invasive edge.

### 3.2. CD3 Status

Survival analysis revealed a significantly increased risk of CRC-specific death among ever smokers with low CD3 cell density in the tumour centre compared to never smokers (adjusted HR: 2.00; 95% CI: 1.23–3.27). Similar associations were observed for overall survival analysis considering deaths from any cause (adjusted HR: 1.71; 95% CI: 1.14–2.54, Table 2).

**Table 2:** Smoking consumption and colorectal cancer-specific survival and overall survival by CD3 status.

	Never smoker	Ever smoker	Unadjusted			Adjusted^a^			Adjusted + MSI^b^			Adjusted sensitivity analysis^a^			
	Patients/No. of deaths		HR	(95% CI)	*P* value	HR	(95% CI)	*P* value	HR	(95% CI)	*P* value	Patients/No of deaths	HR	(95% CI)	*P* value
CRC-specific survival															
Tumour centre CD3												**Tumour centre CD3**			
**High**	109/33	103/36	1.14	0.71–1.83	0.58	1.06	0.63–1.80	0.80	1.11	0.65–1.90	0.68	219/55	0.91	0.51–1.63	0.78
**Low**	95/30	104/49	1.88	1.19–2.96	**0**.**01**	2.00	1.23–3.27	**0**.**01**	1.97	1.20–3.22	**0**.**01**	183/63	1.99	1.15–3.43	**0**.**01**
***P* for interactions**					**0**.**02**										0.17
**Invasive edge CD3**												**Invasive edge CD3**			
**High**	99/25	95/36	1.62	0.97–2.70	0.06	1.74	0.97–3.12	0.06	1.64	0.91–2.95	0.10	207/52	1.8	0.96–3.38	0.07
**Low**	89/33	89/43	1.52	0.96–2.39	0.07	1.46	0.89–2.41	0.13	1.45	0.88–2.39	0.14	184/56	1.22	0.69–2.17	0.48
***P* for interactions**					**0**.**01**										0.62
**Overall survival**															
**Tumour centre CD3**												**Tumour centre CD3**			
**High**	117/41	122/55	1.41	0.96–2.12	0.09	1.41	0.90–2.22	0.13	1.52	0.96–2.42	0.07	219/76	1.35	0.82–2.22	0.23
**Low**	114/49	121/66	1.57	1.09–2.28	**0**.**02**	1.71	1.15–2.54	**0**.**01**	1.71	1.14–2.54	**0**.**01**	212/92	1.75	1.12–2.72	**0**.**01**
***P* for interactions**					**0**.**01**										0.89
**Invasive edge CD3**												**Invasive edge CD3**			
**High**	107/33	111/52	1.81	1.17–2.80	**0**.**01**	1.97	1.20–3.21	**0**.**01**	1.98	1.20–3.27	**0**.**01**	207/74	2.06	1.22–3.49	**0**.**01**
**Low**	106/50	107/61	1.41	0.97–2.06	0.07	1.43	0.95–2.14	0.09	1.41	0.94–2.12	0.09	184/82	1.27	0.79–2.03	0.32
***P* for interactions**					**0**.**01**										0.25

- Excluded patients with a previous history of CRC, or hereditary-linked CRC from the analysis.

- Unknown tumour centre and maximum immune biomarker status removed.

^a^Adjusted; sex, chemotherapy, age, stage, ECOG status and tumour grade differentiation; Adjusted sensitivity analysis; sex, chemotherapy, age, stage, ECOG status and tumour grade differentiation.

^b^Adjusted + MSI; sex, chemotherapy, age, stage, ECOG status, tumour grade differentiation and MSI.

Bold values indicate statistically significant differences (*P* < 0.05)

In contrast, analysis of CD3 density at the invasive margin showed elevated risks of death among ever compared with never smokers with high CD3 density, although the association was not statistically significant for CRC survival (adjusted HR: 1.74; 95% CI: 0.97–3.12) but was for overall survival (adjusted HR: 1.97; 95% CI: 1.20–3.21, Table 2). These associations remained consistent after adjustment for MSI status, and in sensitivity analysis excluding the first year of follow-up.

### 3.3. CD4 Status

In analyses stratified by CD4 density at the tumour centre, ever smokers had a significantly increased risk of CRC-specific death in the presence of low CD4 density compared to never smokers (adjusted HR: 1.75; 95% CI: 1.08–2.86, Table 3). A similar association was observed for overall survival (adjusted HR: 1.59; 95% CI: 1.07–2.36). Interestingly, an increased risk of death from any cause was also found among ever smokers with high CD4 density at the tumour centre, compared to never smokers (adjusted HR: 1.67; 95% CI: 1.05–2.66); no significant association was observed for CRC specific survival when considering smoking status by high CD4 density at the tumour centre (Table 3). These associations remained consistent after adjustment for MSI status, and in sensitivity analysis excluding the first year of follow-up.

**Table 3:** Smoking consumption and colorectal cancer-specific survival and overall survival by CD4 status.

	Never smoker	Ever smoker	Unadjusted			Adjusted^a^			Adjusted + MSI^b^			Adjusted sensitivity analysis^a^			
	Patients/No. of deaths		HR	(95% CI)	*P* value	HR	(95% CI)	*P* value	HR	(95% CI)	*P* value	Patients/No of deaths	HR	(95% CI)	*P* value
CRC-specific survival															
Tumour centre CD4												**Tumour centre CD4**			
**High**	113/31	116/36	1.15	0.71–1.86	0.56	1.24	0.73–2.11	0.42	1.30	0.74–2.25	0.35	238/58	1.13	0.64–2.00	0.67
**Low**	95/32	92/50	2.00	1.28–3.12	**0**.**01**	1.75	1.08–2.86	**0**.**02**	1.76	1.08–2.88	**0**.**02**	198/61	1.81	1.04–3.16	**0**.**04**
***P* for interactions**					**0.01**										0.16
**Invasive edge CD4**												**Invasive edge CD4**			
**High**	101/27	98/34	1.37	0.83–2.28	0.21	1.39	0.80–2.39	0.23	1.25	0.72–2.18	0.94	208/48	1.24	0.67–2.29	0.49
**Low**	89/30	93/44	1.65	1.04–2.63	**0**.**03**	1.53	0.90–2.59	0.11	1.51	0.89–2.57	0.12	191/57	1.39	0.77–2.51	0.26
***P* for interactions**					**0.01**										0.35
**Overall survival**															
**Tumour centre CD4**												**Tumour centre CD4**			
**High**	118/36	135/55	1.52	0.99–2.31	0.05	1.67	1.05–2.66	**0**.**03**	1.80	1.11–2.93	**0**.**02**	238/76	1.58	0.96–2.61	0.07
**Low**	117/54	109/67	1.61	1.13–2.31	**0**.**01**	1.59	1.07–2.36	**0**.**02**	1.54	1.03–2.29	**0**.**03**	198/93	1.72	1.09–2.69	**0**.**02**
***P* for interactions**					**0.01**										0.94
**Invasive edge CD4**												**Invasive edge CD4**			
**High**	112/38	115/51	1.45	0.95–2.21	0.08	1.42	0.90–2.24	0.12	1.36	0.86–2.17	0.18	208/70	1.49	0.89–2.49	0.12
**Low**	104/45	109/60	1.53	1.04–2.25	**0**.**03**	1.66	1.06–2.59	**0**.**02**	1.63	1.05–2.54	**0**.**03**	191/83	1.42	0.87–2.32	0.15
***P* for interactions**					**0.01**										0.99

- Excluded patients with previous history of CRC, or hereditary linked CRC from the analysis.

- Unknown tumour centre and maximum immune biomarker status removed.

^a^Adjusted; sex, chemotherapy, age, stage, ECOG status and tumour grade differentiation; Adjusted sensitivity analysis; sex, chemotherapy, age, stage, ECOG status and tumour grade differentiation.

^b^Adjusted + MSI; sex, chemotherapy, age, stage, ECOG status, tumour grade differentiation and MSI.

Bold values indicate statistically significant differences (*P* < 0.05)

Analysis of CD4 density at the invasive margin showed elevated risks of death among ever compared with never smokers with low CD4 density, although the association was not statistically significant for CRC-specific survival (adjusted HR: 1.53; 95% CI: 0.90–2.59) but was for overall survival (adjusted HR: 1.66; 95% CI: 1.06–2.59, Table 3). These associations remained consistent after adjustment for MSI status, the results for smoking and overall survival in CC patients according to CD4 density at the invasive margin became attenuated in sensitivity analysis excluding the first year of follow-up.

### 3.4. CD8 Status

In the presence of high CD8 density at the tumour centre, ever smokers had a significantly increased risk of CRC-specific death compared to never smokers (adjusted HR: 1.84; 95% CI: 1.09–3.12, Table 4). A similar association was observed for overall survival (adjusted HR: 2.12; 95% CI: 1.36–3.29). These associations remained consistent after adjustment for MSI status (Table 4) and after excluding deaths within the first year of follow-up, although the association between ever versus never smoking and poorer CRC-specific survival in patients with high CD8 density at the tumour centre was no longer statistically significant (adjusted HR: 1.71; 95% CI: 0.97–3.02, Table 4).

**Table 4:** Smoking consumption and colorectal cancer-specific survival and overall survival by CD8 status.

	Never smoker	Ever smoker	Unadjusted			Adjusted^a^			Adjusted + MSI^b^			Adjusted sensitivity analysis^a^			
	Patients/No. of deaths		HR	(95% CI)	*P* value	HR	(95% CI)	*P* value	HR	(95% CI)	*P* value	Patients/No of deaths	HR	(95% CI)	*P* value
CRC-specific survival															
Tumour centre CD8												**Tumour centre CD8**			
**High**	109/29	102/40	1.57	0.97–2.54	0.06	1.84	1.09–3.12	**0**.**02**	1.84	1.06–3.18	**0**.**03**	226/56	1.71	0.97–3.02	0.06
**Low**	99/34	108/46	1.47	0.94–2.29	0.09	1.20	0.73–1.97	0.47	1.21	0.73–2.00	0.45	212/63	1.19	0.68–2.06	0.53
***P* for interactions**					**0**.**01**										0.77
**Invasive edge CD8**												**Invasive edge CD8**			
**High**	102/25	101/32	1.29	0.76–2.18	0.34	1.30	0.73–2.30	0.36	1.23	0.69–2.18	0.48	211/45	1.32	0.69–2.49	0.39
**Low**	93/35	100/49	1.63	1.05–2.51	**0**.**03**	1.66	1.01–2.71	**0**.**04**	1.75	1.05–2.92	**0**.**03**	201/66	1.36	0.79–2.33	0.26
***P* for interactions**					**0**.**02**										0.56
**Overall survival**															
**Tumour centre CD8**												**Tumour centre CD8**			
**High**	115/50	123/61	1.74	1.16–2.59	**0**.**01**	2.12	1.36–3.29	**0**.**01**	2.30	1.44–3.69	**0**.**01**	226/84	1.99	1.24–3.19	**0**.**01**
**Low**	120/40	123/61	1.34	0.92–1.95	0.12	1.15	0.75–1.75	0.51	1.15	0.76–1.76	0.49	212/85	1.25	0.77–2.01	0.35
***P* for interactions**					**0**.**01**										0.44
**Invasive edge CD8**												**Invasive edge CD8**			
**High**	106/29	120/51	1.79	1.13–2.82	**0**.**01**	1.92	1.17–3.14	**0**.**01**	1.91	1.16–3.14	**0**.**01**	211/65	2.09	1.21–3.60	**0**.**01**
**Low**	113/55	115/64	1.36	0.94–1.95	0.09	1.32	0.89–1.97	0.16	1.35	0.90–2.05	0.14	201/92	1.15	0.73–1.80	0.53
***P* for interactions**					**0**.**01**										0.25

–Excluded patients with previous history of CRC, or hereditary linked CRC from the analysis.

–Unknown tumour centre and maximum immune biomarker status removed.

^a^Adjusted; sex, chemotherapy, age, stage, ECOG status, and tumour grade differentiation; Adjusted sensitivity analysis; sex, chemotherapy, age, stage, ECOG status, and tumour grade differentiation.

^b^Adjusted + MSI; sex, chemotherapy, age, stage, ECOG status, tumour grade differentiation, and MSI.

Bold values indicate statistically significant differences (*P* < 0.05)

A significantly increased risk of CRC-specific death was also found among ever smokers compared to never smokers in patients with low CD8 density at the invasive margin (adjusted HR: 1.66; 95% CI: 1.01–2.71). In contrast, a significantly increased risk of death from any cause was observed among ever smokers compared to never smokers in patients with high CD8 density at the invasive margin (adjusted HR: 1.92; 95% CI: 1.17–3.14, Table 4). These associations remained after adjustment for MSI status. In sensitivity analysis excluding the first year of follow-up, the association between smoking status and poorer CRC-specific survival in patients with low CD8 density at the invasive margin was no longer observed, although poorer overall survival in patients with high CD8 density at the invasive margin remained.

### 3.5. FOXP3 status

Survival analysis revealed that ever smokers had a non-significantly increased risk of CRC-specific death compared to never smokers in patients with low FOXP3 density at the tumour centre (adjusted HR: 1.57; 95% CI: 0.96–2.55, Table 5). A significant association for poorer overall survival in ever versus never smokers was observed in both high and low strata of FOXP3 density at the tumour centre.

**Table 5:** Smoking consumption and colorectal cancer-specific survival and overall survival by FOXP3 status.

	Never smoker	Ever smoker	Unadjusted			Adjusted^a^			Adjusted + MSI^b^			Adjusted sensitivity analysis^a^			
	Patients/No. of deaths		HR	(95% CI)	*P* value	HR	(95% CI)	*P* value	HR	(95% CI)	*P* value	Patients/No of deaths	HR	(95% CI)	*P* value
CRC-specific survival															
Tumour centre FOXP3												**Tumour centre FOXP3**			
**High**	103/31	104/39	1.34	0.84–2.16	0.21	1.32	0.78–2.24	0.29	1.40	0.81–2.39	0.22	216/55	1.04	0.58–1.85	0.88
**Low**	104/32	104/46	**1**.**62**	**1.03–2.55**	**0**.**03**	1.57	0.96–2.55	0.07	1.52	0.93–2.48	0.09	218/63	1.63	0.96–2.78	0.07
***P* for interactions**					**0**.**02**										0.64
**Invasive edge FOXP3**												**Invasive edge FOXP3**			
**High**	102/26	86/31	1.48	0.88–2.50	0.14	1.53	0.89–2.65	0.12	1.57	0.89–2.75	0.12	199/47	1.57	0.86–2.86	0.13
**Low**	80/28	100/49	1.72	1.08–2.74	**0**.**03**	1.84	1.11–3.06	**0**.**02**	2.10	1.23–3.57	**0**.**01**	186/59	1.62	0.93–2.82	0.88
***P* for interactions**					**0**.**02**										0.93
**Overall survival**															
**Tumour centre FOXP3**												**Tumour centre FOXP3**			
**High**	113/41	122/57	1.52	1.01–2.27	**0**.**04**	1.59	1.02–2.49	**0**.**04**	1.69	1.07–2.67	**0**.**02**	216/79	1.37	0.85–2.23	0.19
**Low**	118/46	122/64	1.58	1.08–2.31	**0**.**02**	1.59	1.05–2.38	**0**.**02**	1.54	1.03–2.32	**0**.**04**	218/88	1.67	1.06–2.62	**0**.**03**
***P* for interactions**					**0**.**01**										0.67
**Invasive edge FOXP3**												**Invasive edge FOXP3**			
**High**	112/36	100/45	1.61	1.03–2.50	**0**.**03**	1.86	1.17–2.9	**0**.**01**	1.85	1.15–2.97	**0**.**01**	199/68	2.13	1.28–3.56	**0**.**01**
**Low**	94/42	117/66	1.55	1.05–2.29	**0**.**03**	1.66	1.09–2.54	**0**.**02**	1.89	1.21–2.93	**0**.**01**	186/83	1.47	0.92–2.35	0.10
***P* for interactions**					**0**.**01**										0.41

- Excluded patients with a previous history of CRC, or hereditary-linked CRC from the analysis.

- Unknown tumour centre and maximum immune biomarker status removed.

^a^Adjusted; sex, chemotherapy, age, stage, ECOG status, and tumour grade differentiation; Adjusted sensitivity analysis; sex, chemotherapy, age, stage, ECOG status, and tumour grade differentiation.

^b^Adjusted + MSI; sex, chemotherapy, age, stage, ECOG status, tumour grade differentiation, and MSI.

Bold values indicate statistically significant differences (*P* < 0.05)

Compared with never smokers, ever smokers with low FOXP3 density at the invasive margin had a significantly increased risk of CRC-specific death (adjusted HR: 1.84; 95% CI: 1.11–3.06). Again, significant associations for poorer overall survival in ever versus never smokers were observed in both high and low strata of FOXP3 density at the invasive edge.

Adjustment for MSI status either strengthened or did not change the significant associations observed for smoking status and survival according to FOXP3 density. However, after excluding deaths within the first year of follow-up, most associations between FOXP3 density and CRC-specific survival became nonsignificant (Table 5). In contrast, some associations for overall survival persisted. Notably, an increased risk of overall death in ever versus never smokers remained in patients with low FOXP3 density at the tumour centre (adjusted HR: 1.67; 95% CI: 1.06–2.62).

### 3.6. Alcohol consumption and survival analysis by immune biomarkers

**Figure 2:**
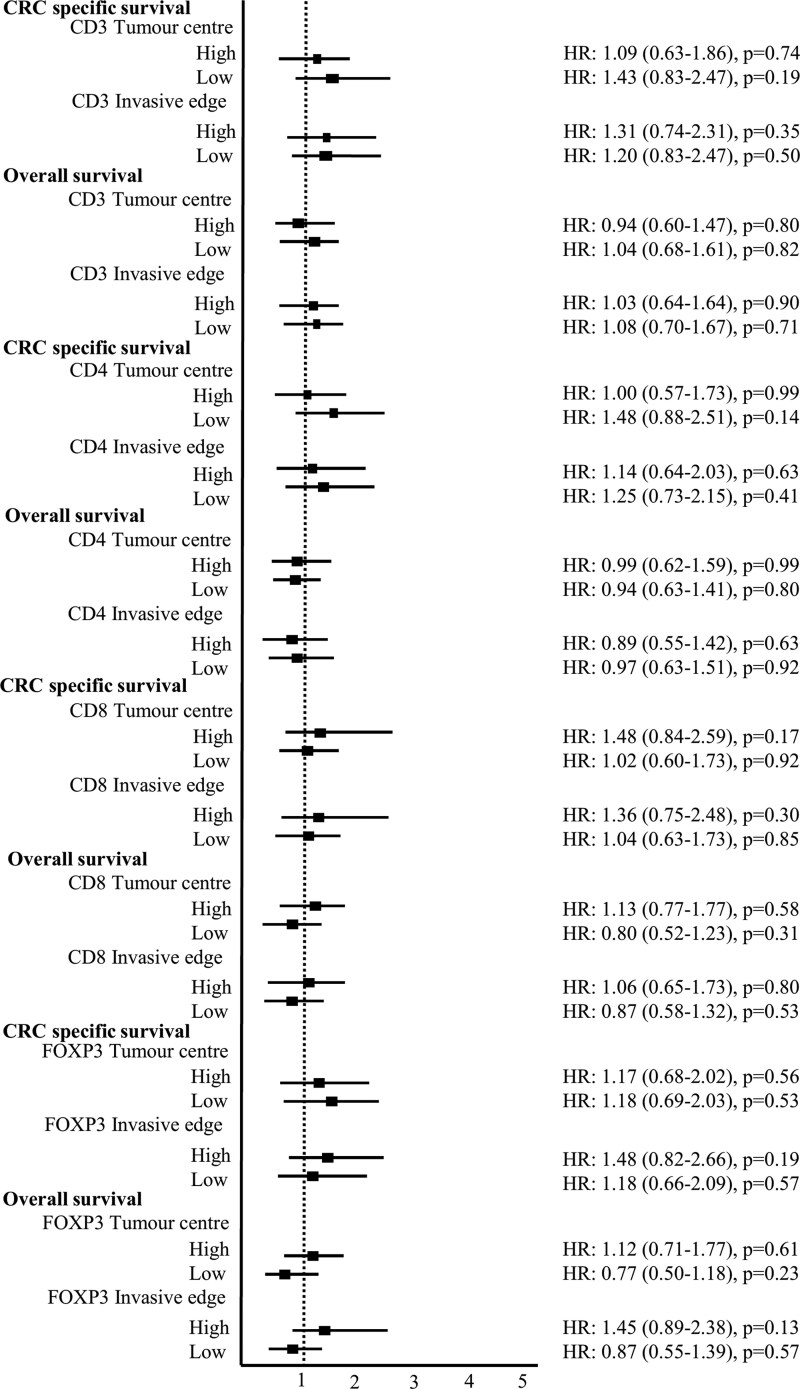
Alcohol and colorectal cancer-specific and overall survival by immune biomarker status at tumour centre and invasive edge.

Supplementary Tables S6–S9 present the associations between ever versus never alcohol consumption and survival outcomes among stage II and III CC patients, stratified by immune biomarker status. No statistically significant associations were observed between alcohol intake and CRC-specific or overall survival across CD3, CD4, CD8, or FOXP3 expression levels, whether assessed at the tumour centre or invasive edge. These null associations remained unchanged after adjustment for MSI status and in sensitivity analyses excluding patients who died within the first year of follow-up. Figure 2 presents the forest plot of the association between ever alcohol and CRC-specific and overall survival, stratified by immune biomarker density at the tumour centre and invasive edge.

## 4. Discussion

In this population-based cohort of patients with stage II and III colon cancer, we identified novel associations between smoking, alcohol consumption, and survival outcomes in relation to immune biomarker expression. Smoking was generally associated with poorer prognosis, with the strongest effects observed among patients exhibiting low CD3 or CD4 but high CD8 densities at the tumour centre. Associations at the invasive margin were less consistent; although ever smokers tended to have increased risks of death, these did not consistently reach statistical significance. No significant relationships were observed between alcohol intake and survival, regardless of immune biomarker expression.

A comprehensive systematic review and meta-analysis by Idos et al. (2020) assessed the prognostic implications of TILs in CRC survival [16]. Their pooled hazard ratios demonstrated that high densities of CD3+ T cells at the tumour centre and at the invasive margin were significantly associated with improved overall, CRC-specific, and disease-free survival, with the strongest effect observed for disease-free survival. Consistent with these findings, our current analysis showed that high compared with low CD3 + status at the tumour centre was significantly associated with improved CRC-specific and overall survival.

Idos et al. also found that high CD8+ T cell density at the tumour centre was significantly associated with improved CRC-specific and disease-free survival, while CD8 + infiltration at the invasive margin did not show a statistically significant association [16]. In line with this, we observed that high CD8 + status was significantly associated with better CRC-specific and overall survival in our study population, however this was observed for both tumour centre and invasive edge CD8 + density.

In contrast, Idos *et al*. reported that high FOXP3 + cell infiltration at the tumour centre was significantly associated with improved CRC-specific, overall and disease-free survival [16]. However, in our study, high FOXP3 + density at the tumour centre was not significantly associated with improved survival outcomes. Nevertheless, we found that high FOXP3 + status at the invasive margin was associated with a decreased risk of CRC-specific and overall death, in agreement with the systematic review findings.

Despite these largely protective associations for high immune biomarker expression and CRC survival observed in our study and previous meta-analyses [16], we identify important new findings that patients who ever smoked had significantly increased risks of CRC-death if CD8 + density was high at the tumour centre.

However, ever smokers had significantly increased risks of CRC-death in low strata of CD3 + or CD4 + density at the tumour centre. These findings suggest the presence of an important nuance in tumour immunology and host-environment interactions, and while some insights may be extrapolated from studies of CRC risk, it is difficult to interpret these associations given that we are the first to report on these findings in relation to survival.

Hamada *et al*. (2019) reported that tobacco smoking significantly increases CRC risk, particularly among individuals with weaker T-cell responses [17]. Their findings suggested that smoking may exert a suppressive effect on T-cell–mediated immunity and highlighted a potential interaction between immune cell density and smoking status in colorectal carcinogenesis [17]. Importantly, their study demonstrated that, compared to never smokers, CRC patients with low CD3 + density had a 38% higher risk of CRC if they were former smokers and a 59% higher risk if they were current smokers. In contrast, no significant association was observed between smoking status and CRC risk among patients with high CD3 + density, suggesting a potential protective effect of a robust T-cell response. Although these findings clearly indicate an interaction between smoking and CD3+ T-cell density, further research is essential to clarify the biological mechanisms underlying this relationship [17]. Nevertheless, the biological mechanisms underlying smoking-induced immune modulation remain unclear [18].

In the current study population, the association between alcohol consumption, immune biomarkers, and survival outcomes in CC patients was evaluated separately for each immune marker, but no significant associations were identified. Although there is strong scientific evidence linking alcohol intake to cancers of the liver, breast, upper aerodigestive tract, pancreas, and colon, it remains unclear whether alcohol primarily contributes to cancer initiation or to disease progression [19]. This is despite evidence that alcohol has been shown to influence immune function in healthy adults [20]. Given that our study showed consistently null associations between alcohol intake and survival outcomes across all categories of immune biomarker status, personalized lifestyle recommendations regarding alcohol consumption following a stage II or III CC diagnosis, based on immune biomarker profile, may not currently be justified.

To the best of our knowledge, this is the first study to investigate the interactions between smoking, alcohol consumption, and colon cancer survival according to immune biomarker status. A major strength is the use of a precise, semi-automated, and validated digital immunoscoring system, ensuring reproducibility of immune expression data. Adjustment for MSI status, consistent with previous findings, did not alter associations between smoking, TILs, and survival [6]. However, several limitations should be acknowledged. Immune data from the invasive margin were derived from a single TMA core, whereas tumour-centre samples were represented by three cores, potentially limiting the assessment of spatial heterogeneity. Prior CRC studies have shown that TMAs can underestimate immune cell densities, particularly for macrophages and B cells, and that heterogeneity is greater in MSI- and BRAF-mutant tumours [21]. Similarly, in nonsmall cell lung cancer, PD-L1 expression correlated well between TMAs and whole sections, but CD8+ T-cell and macrophage densities varied considerably [22] These factors suggest that our immune marker findings, particularly for invasive margin analyses, should be interpreted with caution.

## 5. Conclusion

This is the first study investigating smoking and alcohol consumption in relation to survival among CC patients according to immune-related biomarker expression. Smoking was associated with poorer survival outcomes, with stronger associations observed in CC patients with low CD3 or CD4 density, yet high CD8 density, at the tumour centre.

Results were inconsistent according to immune biomarker density at the invasive edge. Lastly, no significant associations between alcohol intake and survival outcomes were observed in this CC cohort across any immune biomarker categories. Future studies are warranted to validate these observations.

## Supplementary Material

bgag006_Supplementary_Data

## Data Availability

The data that support the findings of this study are not publicly available due to ethical and legal restrictions related to patient confidentiality. Access to the anonymized dataset may be granted upon reasonable request to the Northern Ireland Cancer Registry and the Northern Ireland Biobank, subject to approval by relevant data governance committees. Requests for data access may be directed to the data custodians via the Northern Ireland Cancer Registry (https://www.qub.ac.uk/research-centres/nicr/) and Northern Ireland Biobank (https://www.nibiobank.org/).
