# Supplementary material for: Smoking, alcohol, and colon cancer survival are modified by immune biomarkers: a population-representative study
Source: Carcinogenesis. 2026 Feb 3;47(1):bgag006. doi: 10.1093/carcin/bgag006 (PMC13017039; doi:10.1093/carcin/bgag006)
Supplement: bgag006_Supplementary_Data [file bgag006_supplementary_data.docx]

**Supplementary Tables**

Supplementary Table 1 Median values for positive cells per mm2 of immune biomarkers at the tumour centre and invasive edge.

Supplementary Table 2 Descriptive characteristics of the EPI700 cohort by CD3 immune marker positive score in tumour centre and CD3 invasive edge.

Supplementary Table 3 Descriptive characteristics of the EPI700 cohort by CD4 immune marker. positive score in tumour centre and CD4 invasive edge

Supplementary Table 4 Descriptive characteristics of the EPI700 cohort by CD8 immune marker positive score in tumour centre and CD8 invasive edge.

Supplementary Table 5 Descriptive characteristics of the EPI700 cohort by FOXP3 immune marker positive score in tumour centre and FOXP3 invasive edge

Supplementary Table 6 Alcohol consumption and colorectal cancer-specific survival and overall survival by CD3 status.

Supplementary Table 7 Alcohol consumption and colorectal cancer-specific survival and overall survival by CD4 status.

Supplementary Table 8 Alcohol consumption and colorectal cancer-specific survival and overall survival by CD8 status.

Supplementary Table 9 Alcohol consumption and colorectal cancer-specific survival and overall survival by FOXP3 status.

**Supplementary file**

**Patient characteristics**

Patients with a previous CRC history and inherited CRC were excluded from the EPI700 cohort analysis for this study. In addition, where a patient had missing values for tumour centre, they were removed from that specific analysis; this corresponded to the following number of patients for each immune biomarker investigated: CD3 tumour centre n=14, CD3 invasive edge n=64, CD4 tumour centre n=8, CD4 invasive edge n=57, CD8 tumour centre n=6, CD8 invasive edge n=38, FOXP3 tumour centre n=14, FOXP3 invasive edge n=79.

The resulting number and proportion of patients remaining for inclusion in analysis were 625 (94.5%) for CD3 density, 631 (95.4%) for CD4 density, 633 (95.7%) for CD8 density, 625 (94.5%) for FOXP3 density number positive cells per mm^2^ in tumour centre. For invasive edge the resulting number and proportion of patients remaining for inclusion in analysis were 575 (87%) for CD3 density, 582 (88.1%) for CD4 density, 601 (90.9%) for CD8 density, 560 (84.7%) for FOXP3 density number positive cells per mm^2^. Expression of IHC biomarkers is described as “high” or “low” if above/equal to or below the median value of expression for dichotomisation in analysis.

Patient characteristics for the EPI700 cohort presented that there were no differences in age, sex, stage, tumour location, differentiation grade, adjuvant chemotherapy receipt, family history of CRC or ECOG status according to categories of CD3, CD4, CD8 or FOXP3 expression, with the exception of higher FOXP3 expression being more common in females than males and higher CD8 tumour expression being more common in right-sided and poorly differentiated tumours. We did observe differences in IHC expression status according to year of diagnosis, which likely reflects that TMAs were created according to year. Regardless, all confounders were retained for multivariable models, for consistency with previous research and biological plausibility.

**Statistical analysis**

Multivariable-adjusted Cox models were used to account for potential confounders of CC-specific survival. Covariates included sex; age at diagnosis (<50, 50–<60, 60–<70, 70–<80, >80 years); cancer stage (II or III); tumour differentiation (poorly, well/moderately, or unknown); receipt of adjuvant chemotherapy (yes or no); tumour location (proximal, distal, or unknown); family history of CRC (yes, no, or unknown); ECOG performance status (0–1, 2, 3–4); and MSI status (microsatellite stable [MSS], MSI-high, or unknown).

**Immune biomarkers scoring and assessment**

There is no evidence for the adoption of a specific cut off point for CD3, CD4, CD8, and FOXP3 densities derived from digital immunoscoring. Immune biomarker’s low versus high status was therefore categorised around the median value for survival analyses testing the interaction between alcohol and smoking consumption and each biomarker, in the tumour centre and advancing edge. Supplementary table 1 represented the median values for each immune biomarker.

Supplementary Table 1 Median values for positive cells per mm^2^ of immune biomarkers at the tumour centre and invasive edge.

|  | Tumour centre | Invasive edge |
| --- | --- | --- |
| CD3 positive cells per mm^2^ | 544 | 774 |
| CD4 positive cells per mm^2^ | 588.7 | 828 |
| CD8 positive cells per mm^2^ | 262.8 | 441.9 |
| FOXP3 positive cells per mm^2^ | 104.9 | 152.5 |

Supplementary Table 2 Descriptive characteristics of the EPI700 cohort by CD3 immune marker positive score in tumour centre and CD3 invasive edge.

|  |  | Tumour core CD3 | |  |  | Invasive edge CD3 | |  |
| --- | --- | --- | --- | --- | --- | --- | --- | --- |
| Total | | **High** | **Low** | **P-value** | **Total** | **High** | **Low** | **P-value** |
| n=625 | | **n=307** | **n=318** |  | **n=575** | **n=282** | **n=293** |  |
|  |  | **%= 49.1** | **%= 50.9** |  |  | **%= 49.1** | **%= 50.9** |  |
| *Age category* | | | | | | | | |
| <50 | 33 | 21 (63.6) | 12 (36.4) | 0.512 | 31 | 18 (58.1) | 13 (41.9) | 0.152 |
| 50-<60 | 58 | 26 (44.8) | 32 (55.2) |  | 54 | 31 (57.4) | 23 (42.6) |  |
| 60-<70 | 171 | 84 (49.1) | 87 (50.9) |  | 150 | 75 (50.0) | 75 (50.0) |  |
| 70-<80 | 228 | 111 (48.7) | 117 (51.5) |  | 213 | 107 (50.2) | 106 (49.8) |  |
| >80 | 135 | 65 (48.2) | 70 (51.9) |  | 127 | 51 (40.2) | 76 (59.8) |  |
| *Gender* | | | | | | | | |
| Female | 284 | 151 (53.2) | 133 (46.8) | 0.065 | 258 | 138 (53.5) | 120 (46.5) | 0.054 |
| Male | 341 | 156 (45.8) | 185 (54.3) |  | 317 | 144 (45.4) | 173 (54.6) |  |
| *Stage* | | | | | | | | |
| II | 375 | 188 (50.1) | 187 (49.9) | 0.535 | 349 | 184 (52.7) | 165 (47.3) | **0.028** |
| III | 250 | 119 (47.6) | 131 (52.4) |  | 226 | 98 (43.4) | 128 (56.6) |  |
| *Year of diagnosis* | | | | | | | | |
| 2004 | 96 | 36 (37.5) | 60 (62.5) | **0.008** | 90 | 39 (43.3) | 51 (56.7) | **0.0001** |
| 2005 | 123 | 53 (43.1) | 70 (56.9) |  | 117 | 39 (33.3) | 78 (66.7) |  |
| 2006 | 122 | 63 (51.6) | 59 (48.4) |  | 120 | 77 (64.2) | 43 (35.8) |  |
| 2007 | 129 | 63 (48.8) | 66 (51.2) |  | 131 | 58 (44.3) | 73 (55.7) |  |
| 2008 | 155 | 92 (59.4) | 63 (40.7) |  | 117 | 69 (59.0) | 48 (41.0) |  |
| *Tumour location* | | | | | | | | |
| Right-sided | 352 | 186 (52.8) | 166 (47.2) | 0.058 | 326 | 163 (50.0) | 163 (50.0) | 0.748 |
| Left-sided | 267 | 117 (43.8) | 150 (56.2) |  | 244 | 116 (47.5) | 128 (52.5) |  |
| Colon uns. | 6 | 4 (66.7) | 2 (33.3) |  | 5 | 3 (60.0) | 2 (40.0) |  |
| *Differentiation grade* | | | | | | | | |
| Well-moderately | 537 | 255 (47.5) | 282 (52.5) | 0.122 | 492 | 234 (47.6) | 258 (52.4) | 0.09 |
| Poorly | 84 | 50 (59.5) | 34 (40.5) |  | 79 | 47 (59.50 | 32 (40.5) |  |
| Unknown | 4 | 2 (50.0) | 2 (50.0) |  | 4 | 1 (25.0) | 3 (75.0) |  |
| *Adjuvant chemotherapy* | | | | | | | | |
| Yes | 174 | 79 (45.4) | 95 (54.6) | 0.248 | 155 | 74 (47.7) | 81 (52.3) | 0.704 |
| No | 451 | 228 (50.6) | 223 (49.5) |  | 420 | 208 (49.5) | 212 (50.5) |  |
| *Family history of CRC* | | | | | | | | |
| Yes | 75 | 40 (53.3) | 35 (46.7) | 0.163 | 66 | 30 (45.5) | 36 (54.6) | 0.056 |
| No | 315 | 163 (51.8) | 152 (48.3) |  | 291 | 157 (54.0) | 134 (46.1) |  |
| Unknown | 235 | 104 (44.3) | 131 (55.7) |  | 218 | 95 (43.6) | 123 (56.4) |  |
| *ECOG Categories* | | | | | | | | |
| 0/1 | 320 | 167 (52.2) | 153 (47.8) | 0.07 | 302 | 153 (50.7) | 149 (49.3) | 0.433 |
| 2 | 38 | 13 (34.2) | 25 (65.8) |  | 36 | 18 (50.0) | 18 (50.0) |  |
| 3/4 | 29 | 10 (34.5) | 19 (65.5) |  | 24 | 8 (33.3) | 16 (66.7) |  |
| Unknown | 238 | 117 (49.2) | 121 (50.8) |  | 213 | 103 (48.4) | 110 (51.6) |  |

-Excluded patients with previous history of colorectal cancer, or genetically linked colorectal cancer condition form the analysis.

-Unknown tumour core and maximum CD3 status removed

Supplementary Table 3 Descriptive characteristics of the EPI700 cohort by CD4 immune marker. positive score in tumour centre and CD4 invasive edge

|  |  | Tumour core CD4 | | | |  |  | Invasive edge CD4 | | | |  |
| --- | --- | --- | --- | --- | --- | --- | --- | --- | --- | --- | --- | --- |
| Total | | **High** | | **Low** | | **P-value** | **Total** | **High** | | **Low** | | **P-value** |
| n=631 | | **n=316** | | **n=315** | |  | **n=582** | **n=288** | | **n=294** | |  |
|  |  | **%= 50.1** | | **%= 49.9** | |  |  | **%= 49.5** | | **%= 50.5** | |  |
| *Age category* | | | | | | | | | | | | |
| <50 | 34 | 22 (64.7) | | 12 (35.3) | | 0.19 | 32 | 17 (53.1) | | 15 (46.0) | | 0.797 |
| 50-<60 | 60 | 33 (55.0) | | 27 (45.0) | |  | 53 | 30 (56.6) | | 23 (43.40 | |  |
| 60-<70 | 171 | 84 (49.1) | | 87 (50.9) | |  | 154 | 77 (50.0) | | 77 (50.0) | |  |
| 70-<80 | 230 | 118 (51.3) | | 112 (48.7) | |  | 214 | 103 (48.1) | | 111 (51.9) | |  |
| >80 | 136 | 59 (43.4) | | 77 (56.6) | |  | 129 | 61 (47.3) | | 68 (52.7) | |  |
| *Gender* |  |  |  |  |  |  |  |  |  |  |  |  |
| Female | 288 | 150 (52.1) | | 138 (47.9) | | 0.356 | 260 | 134 (51.5) | | 126 (48.5) | | 0.373 |
| Male | 343 | 166 (48.4) | | 177 (51.6) | |  | 322 | 154 (47.8) | | 168 (52.2) | |  |
| *Stage* |  |  |  |  |  |  |  |  |  |  |  |  |
| II | 378 | 194 (51.3) | | 184 (48.7) | | 0.445 | 353 | 185 (52.4) | | 168 (47.6) | | **0.08** |
| III | 253 | 122 (48.2) | | 131 (51.8) | |  | 229 | 103 (45.0) | | 126 (55.0) | |  |
| *Year of diagnosis* | | | | | | | | | | | | |
| 2004 | 94 | 25 (26.0) | | 71 (74.0) | | **0.0001** | 90 | 10 (11.1) | | 80 (88.9) | | **0.0001** |
| 2005 | 124 | 42 (33.9) | | 82 (66.1) | |  | 119 | 32 (26.9) | | 87 (73.1) | |  |
| 2006 | 123 | 65 (52.9) | | 58 (47.2) | |  | 116 | 80 (69.0) | | 36 (31.0) | |  |
| 2007 | 133 | 53 (39.9) | | 80 (60.2) | |  | 131 | 66 (50.4) | | 65 (49.6) | |  |
| 2008 | 155 | 131 (84.5) | | 24 (15.5) | |  | 126 | 100 (79.4) | | 26 (20.6) | |  |
| *Tumour location* | | | | | | | | | | | | |
| Right-sided | 356 | 165 (46.4) | | 191 (53.7) | | 0.087 | 336 | 158 (47.0) | | 178 (53.0) | | 0.18 |
| Left-sided | 269 | 147 (54.7) | | 122 (45.4) | |  | 241 | 126 (52.3) | | 115 (47.7) | |  |
| Colon uns. | 6 | 4 (66.7) | | 2 (33.3) | |  | 5 | 4 (80.0) | | 1 (20.0) | |  |
| *Differentiation grade* | | | | | | | | | | | | |
| Well-moderately | 541 | 267 (49.4) | | 274 (50.7) | | 0.324 | 501 | 245 (48.9) | | 256 (51.1) | | 0.403 |
| Poorly | 86 | 48 (55.8) | | 38 (44.2) | |  | 77 | 42 (54.6) | | 35 (45.5) | |  |
| Unknown | 4 | 1 (25.0) | | 3 (75.0) | |  | 4 | 1 (25.0) | | 3 (75.0) | |  |
| *Adjuvant chemotherapy* | | | | | | | | | | | | |
| Yes | 177 | 94 (53.1) | | 83 (46.9) | | 0.342 | 157 | 76 (48.4) | | 81 (51.6) | | 0.752 |
| No | 454 | 222 (48.9) | | 232 (51.1) | |  | 425 | 212 (49.9) | | 213 (50.1) | |  |
| *Family history of CRC* | | | | | | | | | | | | |
| Yes | 76 | 40 (52.6) | | 36 (47.4) | | 0.402 | 68 | 32 (47.1) | | 36 (52.9) | | 0.155 |
| No | 319 | 166 (52.0) | | 153 (48.0) | |  | 294 | 157 (53.4) | | 137 (46.6) | |  |
| Unknown | 236 | 110 (46.6) | | 126 (53.4) | |  | 220 | 99 (45.0) | | 121 (55.0) | |  |
| *ECOG Categories* | | | | | | | | | | | | |
| 0/1 | 325 | 160 (49.2) | | 165 (50.8) | | 0.498 | 300 | 151 (50.3) | | 149 (49.7) | | 0.919 |
| 2 | 38 | 20 (52.6) | | 18 (47.4) | |  | 36 | 19 (52.8) | | 17 (47.2) | |  |
| 3/4 | 29 | 11 (37.9) | | 18 (62.1) | |  | 28 | 13 (46.4) | | 15 (53.6) | |  |
| Unknown | 239 | 125 (52.3) | | 114 (47.7) | |  | 218 | 105 (48.2) | | 113 (51.8) | |  |

-Excluded patients with previous history of colorectal cancer, or genetically linked colorectal cancer condition form the analysis.

-Unknown tumour core and maximum CD4 status removed

Supplementary Table 4 Descriptive characteristics of the EPI700 cohort by CD8 immune marker positive score in tumour centre and CD8 invasive edge.

|  |  | Tumour core CD8 | |  |  | Invasive edge CD8 | |  |
| --- | --- | --- | --- | --- | --- | --- | --- | --- |
| Total | | **High** | **Low** | **P-value** | **Total** | **High** | **Low** | **P-value** |
| n=633 | | **n=314** | **n=319** |  | **n=601** | **n=295** | **n=306** |  |
|  |  | **%= 49.6** | **%= 50.4** |  |  | **%= 49.1** | **%= 50.9** |  |
| *Age category* | | | | | | | | |
| <50 | 34 | 19 (55.9) | 15 (44.1) | 0.153 | 34 | 17 (50.0) | 17 (50.0) | 0.266 |
| 50-<60 | 60 | 24 (40.0) | 36 (60.0) |  | 56 | 28 (50.0) | 28 (50.0) |  |
| 60-<70 | 171 | 76 (44.4) | 95 (55.6) |  | 161 | 87 (54.0) | 74 (46.0) |  |
| 70-<80 | 232 | 126 (54.3) | 106 (45.7) |  | 218 | 109 (50.0) | 109 (50.0) |  |
| >80 | 136 | 69 (50.7) | 67 (49.3) |  | 132 | 54 (40.9) | 78 (59.1) |  |
| *Gender* | | | | | | | | |
| Female | 289 | 155 (53.6) | 134 (46.4) | 0.063 | 270 | 131 (48.5) | 139 (51.5) | 0.802 |
| Male | 344 | 159 (46.2) | 185 (53.8) |  | 331 | 164 (49.6) | 167 (50.5) |  |
| *Stage* | | | | | | | | |
| II | 378 | 196 (51.9) | 182 (48.2) | 0.169 | 360 | 183 (50.8) | 177 (49.2) | 0.295 |
| III | 255 | 118 (46.3) | 137 (53.7) |  | 241 | 112 (46.5) | 129 (53.5) |  |
| *Year of diagnosis* | | | | | | | | |
| 2004 | 96 | 52 (54.2) | 44 (45.80 | 0.331 | 82 | 31 (37.8) | 51 (62.2) | **0.001** |
| 2005 | 124 | 54 (43.6) | 70 (56.5) |  | 111 | 46 (41.4) | 65 (58.6) |  |
| 2006 | 124 | 66 (53.2) | 58 (46.8) |  | 122 | 69 (56.6) | 53 (43.4) |  |
| 2007 | 134 | 61 (45.5) | 73 (54.5) |  | 133 | 57 (42.9) | 76 (57.1) |  |
| 2008 | 155 | 81 (52.3) | 74 (47.7) |  | 153 | 92 (60.1) | 61 (39.9) |  |
| *Tumour location* | | | | | | | | |
| Right-sided | 357 | 197 (55.2) | 160 (44.8) | **0.006** | 340 | 166 (48.8) | 174 (51.2) | 0.241 |
| Left-sided | 270 | 115 (42.6) | 155 (57.4) |  | 255 | 124 (48.6) | 131 (51.4) |  |
| Colon uns. | 6 | 2 (33.3) | 4 (6.7) |  | 6 | 5 (83.3) | 1 (16.7) |  |
| *Differentiation grade* | | | | | | | | |
| Well-moderately | 542 | 251 (46.3) | 291 (53.7) | **0.001** | 514 | 246 (47.9) | 268 (52.1) | 0.151 |
| Poorly | 87 | 61 (70.1) | 26 (29.9) |  | 83 | 48 (57.8) | 35 (42.2) |  |
| Unknown | 4 | 2 (50.0) | 2 (50.0) |  | 4 | 1 (25.0) | 3 (75.0) |  |
| *Adjuvant chemotherapy* | | | | | | | | |
| Yes | 177 | 81 (45.8) | 96 (54.2) | 0.228 | 166 | 86 (51.8) | 80 (48.2) | 0.41 |
| No | 456 | 233 (51.1) | 223 (48.9) |  | 435 | 209 (48.1) | 226 (52.0) |  |
| *Family history of CRC* | | | | | | | | |
| Yes | 76 | 33 (43.4) | 43 (56.6) | 0.301 | 71 | 34 (47.9) | 37 (52.1) | 0.785 |
| No | 321 | 168 (52.3) | 153 (47.7) |  | 301 | 152 (50.5) | 149 (49.5) |  |
| Unknown | 236 | 113 (47.9) | 123 (52.1) |  | 229 | 109 (47.6) | 120 (52.4) |  |
| *ECOG Categories* | | | | | | | | |
| 0/1 | 326 | 171 (52.5) | 155 (47.6) | 0.223 | 311 | 152 (48.9) | 159 (51.1) | 0.532 |
| 2 | 39 | 17 (43.6) | 22 (56.4) |  | 38 | 21 (55.3) | 17 (44.7) |  |
| 3/4 | 29 | 10 (34.5) | 19 (65.5) |  | 27 | 10 (37.0) | 17 (63.0) |  |
| Unknown | 239 | 116 (48.5) | 123 (51.5) |  | 225 | 112 (49.8) | 113 (50.2) |  |

-Excluded patients with previous history of colorectal cancer, or genetically linked colorectal cancer condition form the analysis.

-Unknown tumour core and maximum CD8 status removed

Supplementary Table 5 Descriptive characteristics of the EPI700 cohort by FOXP3 immune marker positive score in tumour centre and FOXP3 invasive edge.

|  |  | Tumour core FOXP3 | |  |  | Invasive edge FOXP3 | |  |
| --- | --- | --- | --- | --- | --- | --- | --- | --- |
| Total | | **High** | **Low** | **P-value** | **Total** | **High** | **Low** | **P-value** |
| n=625 | | **n=307** | **n=318** |  | **n=560** | **n=272** | **n=282** |  |
|  |  | **%= 49.1** | **%= 50.9** |  |  | **%= 49.7** | **%= 50.4** |  |
| *Age category* | | | | | | | | |
| <50 | 34 | 21 (61.8) | 13 (38.2) | 0.671 | 32 | 13 (40.6) | 19 (59.4) | 0.521 |
| 50-<60 | 59 | 28 (47.5) | 31 (52.5) |  | 52 | 29 (55.8) | 23 (44.2) |  |
| 60-<70 | 171 | 82 (48.0) | 89 (52.1) |  | 143 | 67 (46.9) | 76 (53.2) |  |
| 70-<80 | 227 | 111 (48.9) | 116 (51.1) |  | 207 | 109 (52.70 | 98 (47.3) |  |
| >80 | 134 | 67 (50.0) | 67 (50.0) |  | 126 | 60 (47.6) | 66 (52.4) |  |
| *Gender* | | | | | | | | |
| Female | 284 | 153 (53.9) | 131 (46.1) | **0.043** | 253 | 152 (60.1) | 101 (39.9) | **0.0001** |
| Male | 341 | 156 (45.8) | 185 (54.3) |  | 307 | 126 (41.0) | 181 (59.0) |  |
| *Stage* | | | | | | | | |
| II | 375 | 196 (52.3) | 179 (47.7) | 0.083 | 335 | 177 (52.8) | 158 (47.2) | 0.065 |
| III | 250 | 113 (45.2) | 137 (54.8) |  | 225 | 101 (44.9) | 124 (55.1) |  |
| *Year of diagnosis* | | | | | | | | |
| 2004 | 95 | 57 (60.0) | 38 (40.0) | **0.0001** | 92 | 63 (68.5) | 29 (31.5) | **0.0001** |
| 2005 | 124 | 37 (29.8) | 87 (70.2) |  | 109 | 27 (24.8) | 82 (75.2) |  |
| 2006 | 123 | 49 (39.8) | 74 (60.2) |  | 112 | 65 (58.0) | 47 (42.0) |  |
| 2007 | 128 | 47 (36.7) | 81 (63.3) |  | 128 | 48 (37.5) | 80 (62.5) |  |
| 2008 | 155 | 119 (76.8) | 36 (23.2) |  | 119 | 75 (63.0) | 44 (37.0) |  |
| *Tumour location* | | | | | | | | |
| Right-sided | 354 | 182 (51.4) | 172 (48.6) | 0.423 | 322 | 163 (50.6) | 159 (49.4) | 0.752 |
| Left-sided | 265 | 125 (47.2) | 140 (52.8) |  | 233 | 112 (48.1) | 121 (51.9) |  |
| Colon uns. | 6 | 2 (33.3) | 4 (66.7) |  | 5 | 3 (60.0) | 2 (40.0) |  |
| *Differentiation grade* | | | | | | | | |
| Well-moderately | 536 | 263 (49.1) | 273 (50.9) | 0.496 | 484 | 242 (50.0) | 242 (50.0) | 0.807 |
| Poorly | 85 | 45 (52.9) | 40 (47.1) |  | 73 | 35 (48.0) | 38 (52.1) |  |
| Unknown | 4 | 1 (25.0) | 3 (75.0) |  | 3 | 1 (33.3) | 2 (66.7) |  |
| *Adjuvant chemotherapy* | | | | | | | | |
| Yes | 175 | 81 (46.3) | 94 (53.7) | 0.325 | 156 | 80 (51.3) | 76 (48.7) | 0.63 |
| No | 450 | 228 (50.67) | 222 (49.3) |  | 404 | 198 (49.0) | 206 (51.0) |  |
| *Family history of CRC* | | | | | | | | |
| Yes | 75 | 40 (53.3) | 35 (46.7) | 0.77 | 65 | 36 (55.4) | 29 (44.6) | 0.429 |
| No | 316 | 155 (49.1) | 161 (51.0) |  | 285 | 144 (50.5) | 141 (49.5) |  |
| Unknown | 234 | 114 (48.7) | 120 (51.3) |  | 210 | 98 (46.7) | 112 (53.3) |  |
| *ECOG Categories* | | | | | | | | |
| 0/1 | 321 | 168 (52.3) | 153 (47.7) | 0.165 | 293 | 145 (49.5) | 148 (50.5) | 0.817 |
| 2 | 37 | 21 (56.8) | 16 (43.2) |  | 33 | 19 (57.6) | 14 (42.4) |  |
| 3/4 | 28 | 10 (35.7) | 18 (64.3) |  | 26 | 13 (50.0) | 13 (50.0) |  |
| Unknown | 239 | 110 (46.0) | 129 (54.0) |  | 208 | 101 (48.6) | 107 (51.4) |  |

-Excluded patients with previous history of colorectal cancer, or genetically linked colorectal cancer condition form the analysis.

-Unknown tumour core and maximum FOXP3 status removed

Supplementary Table 6 Alcohol consumption and colorectal cancer-specific survival and overall survival by CD3 status.

| CRC-specific survival | | | | | | | | | | | | | | | |
| --- | --- | --- | --- | --- | --- | --- | --- | --- | --- | --- | --- | --- | --- | --- | --- |
|  | **Never alcohol** | **Ever alcohol** | **Unadjusted** | | | **Adjusted*** | | | **Adjusted+MSI** | | | **Adjusted sensitivity analysis *** | | | |
|  | **Patients/ No. of deaths** | | **HR** | **(95% CI)** | **P value** | **HR** | **(95% CI)** | **P value** | **HR** | **(95% CI)** | **P value** | **Patients/No of deaths** | **HR** | **(95% CI)** | **P value** |
| Tumour centre CD3 |  |  |  |  |  |  |  |  |  |  |  | **Tumour centre CD3** |  |  |  |
| High | 75/24 | 128/39 | 1.02 | 0.61-1.70 | 0.92 | 1.09 | 0.63-1.86 | 0.748 | 1.09 | 0.63-1.86 | 0.75 | 212/52 | 1.15 | 0.64-2.09 | 0.63 |
| Low | 64/25 | 114/46 | 1.23 | 0.75-2.00 | 0.40 | 1.43 | 0.83-2.47 | 0.191 | 1.47 | 0.85-2.55 | 0.16 | 192/58 | 1.29 | 0.71-2.34 | 0.40 |
| p for interactions |  |  |  |  | 0.50 |  |  |  |  |  |  |  |  |  | 0.88 |
| Invasive edge CD3 |  |  |  |  |  |  |  |  |  |  |  | **Invasive edge CD3** |  |  |  |
| High | 66/21 | 117/35 | 1.05 | 0.61-1.81 | 0.84 | 1.31 | 0.74-2.31 | 0.344 | 1.31 | 0.74-2.31 | 0.34 | 196/48 | 1.29 | 0.70-2.37 | 0.40 |
| Low | 63/24 | 98/45 | 1.34 | 0.82-2.21 | 0.24 | 1.20 | 0.70-2.06 | 0.503 | 1.25 | 0.72-2.15 | 0.41 | 170/54 | 1.04 | 0.57-1.90 | 0.88 |
| p for interactions |  |  |  |  | 0.30 |  |  |  |  |  |  |  |  |  | 0.55 |
| Overall survival | | | | | | | | | | | | | | | |
| Tumour centre CD3 |  |  |  |  |  |  |  |  |  |  |  | **Tumour centre CD3** |  |  |  |
| High | 89/38 | 140/51 | 0.84 | 0.55-1.29 | 0.45 | 0.94 | 0.60-1.47 | 0.797 | 0.94 | 0.60-1.47 | 0.81 | 212/72 | 0.98 | 0.60-1.62 | 0.97 |
| Low | 84/45 | 128/60 | 0.89 | 0.60-1.31 | 0.57 | 1.04 | 0.68-1.61 | 0.825 | 1.05 | 0.68-1.63 | 0.79 | 192/85 | 1.07 | 0.66-1.73 | 0.76 |
| p for interactions |  |  |  |  | 0.35 |  |  |  |  |  |  |  |  |  | 0.98 |
| Invasive edge CD3 |  |  |  |  |  |  |  |  |  |  |  | **Invasive edge CD3** |  |  |  |
| High | 79/34 | 127/45 | 0.84 | 0.54-1.32 | 0.47 | 1.03 | 0.64-1.64 | 0.90 | 1.05 | 0.66-1.68 | 0.82 | 196/69 | 1.02 | 0.62-1.67 | 0.93 |
| Low | 81/42 | 113/60 | 1.03 | 0.69-1.53 | 0.87 | 1.08 | 0.70-1.67 | 0.711 | 1.12 | 0.72-1.73 | 0.60 | 170/78 | 1.07 | 0.65-1.76 | 0.77 |
| p for interactions |  |  |  |  | 0.70 |  |  |  |  |  |  |  |  |  | 0.30 |
|  |  |  |  |  |  |  |  |  |  |  |  |  |  |  |  |

- Excluded patients with previous history of CRC, or hereditary linked CRC from the analysis.

- Unknown tumour centre and maximum immune biomarker status removed.

*Adjusted; sex, chemotherapy, age, stage, ECOG status and tumour grade differentiation.

**Adjusted+MSI; sex, chemotherapy, age, stage, ECOG status, tumour grade differentiation and MSI.

*Adjusted sensitivity analysis; sex, chemotherapy, age, stage, ECOG status and tumour grade differentiation.

Supplementary Table 7 Alcohol consumption and colorectal cancer-specific survival and overall survival by CD4 status.

| CRC-specific survival | | | | | | | | | | | | | | | |
| --- | --- | --- | --- | --- | --- | --- | --- | --- | --- | --- | --- | --- | --- | --- | --- |
|  | **Never alcohol** | **Ever alcohol** | **Unadjusted** | | | **Adjusted*** | | | **Adjusted+MSI **** | | | **Adjusted sensitivity analysis *** | | | |
|  | **Patients/ No. of deaths** | | **HR** | **(95% CI)** | **P value** | **HR** | **(95% CI)** | **P value** | **HR** | **(95% CI)** | **P value** | **Patients/No of deaths** | **HR** | **(95% CI)** | **P value** |
| Tumour centre CD4 |  |  |  |  |  |  |  |  |  |  |  | **Tumour centre CD4** |  |  |  |
| High | 83/26 | 134/35 | 0.87 | 0.52-1.45 | 0.61 | 1.00 | 0.57-1.73 | 0.99 | 1.03 | 0.59-1.79 | 0.90 | 226/54 | 1.01 | 0.56-1.80 | 0.97 |
| Low | 57/23 | 111/50 | 1.38 | 0.84-2.27 | 0.20 | 1.48 | 0.88-2.51 | 0.14 | 1.55 | 0.91-2.63 | 0.10 | 182/56 | 1.43 | 0.80-2.56 | 0.22 |
| p for interactions |  |  |  |  | 0.55 |  |  |  |  |  |  |  |  |  | 0.38 |
| Invasive edge CD4 |  |  |  |  |  |  |  |  |  |  |  | **Invasive edge CD4** |  |  |  |
| High | 69/22 | 120/35 | 1.01 | 0.59-1.71 | 0.98 | 1.14 | 0.64-2.03 | 0.63 | 1.19 | 0.67-2.10 | 0.55 | 199/46 | 1.11 | 0.59-2.08 | 0.74 |
| Low | 61/22 | 103/45 | 1.40 | 0.83-2.33 | 0.20 | 1.25 | 0.73-2.15 | 0.41 | 1.25 | 0.73-2.16 | 0.41 | 175/54 | 1.26 | 0.69-2.29 | 0.45 |
| p for interactions |  |  |  |  | 0.32 |  |  |  |  |  |  |  |  |  | 0.30 |
| Overall survival | | | | | | | | | | | | | | | |
| Tumour centre CD4 |  |  |  |  |  |  |  |  |  |  |  | **Tumour centre CD4** |  |  |  |
| High | 92/35 | 147/48 | 0.89 | 0.57-1.38 | 0.62 | 0.99 | 0.62-1.59 | 0.986 | 1.02 | 0.64-1.64 | 0.91 | 226/70 | 0.99 | 0.59-1.65 | 0.98 |
| Low | 82/48 | 124/63 | 0.83 | 0.57-1.21 | 0.34 | 0.94 | 0.63-1.41 | 0.802 | 0.98 | 0.65-1.47 | 0.93 | 182/87 | 1.01 | 0.64-1.58 | 0.96 |
| p for interactions |  |  |  |  | 0.29 |  |  |  |  |  |  |  |  |  | 0.91 |
| Invasive edge CD4 |  |  |  |  |  |  |  |  |  |  |  | **Invasive edge CD4** |  |  |  |
| High | 84/37 | 132/47 | 0.77 | 0.50-1.19 | 0.24 | 0.89 | 0.55-1.42 | 0.629 | 0.91 | 0.57-1.45 | 0.70 | 199/67 | 0.93 | 0.55-1.57 | 0.04 |
| Low | 77/38 | 116/58 | 1.05 | 0.69-1.58 | 0.81 | 0.97 | 0.63-1.51 | 0.928 | 0.96 | 0.61-1.49 | 0.86 | 175/78 | 1.02 | 0.63-1.67 | 0.01 |
| p for interactions |  |  |  |  | 0.62 |  |  |  |  |  |  |  |  |  | 0.26 |

- Excluded patients with previous history of CRC, or hereditary linked CRC from the analysis.

- Unknown tumour centre and maximum immune biomarker status removed.

*Adjusted; sex, chemotherapy, age, stage, ECOG status and tumour grade differentiation.

**Adjusted+MSI; sex, chemotherapy, age, stage, ECOG status, tumour grade differentiation and MSI.

*Adjusted sensitivity analysis; sex, chemotherapy, age, stage, ECOG status and tumour grade differentiation.

Supplementary Table 8 Alcohol consumption and colorectal cancer-specific survival and overall survival by CD8 status.

| CRC-specific survival | | | | | | | | | | | | | | | |
| --- | --- | --- | --- | --- | --- | --- | --- | --- | --- | --- | --- | --- | --- | --- | --- |
|  | **Never alcohol** | **Ever alcohol** | **Unadjusted** | | | **Adjusted*** | | | **Adjusted+MSI **** | | | **Adjusted sensitivity analysis *** | | | |
|  | **Patients/ No. of deaths** | | **HR** | **(95% CI)** | **P value** | **HR** | **(95% CI)** | **P value** | **HR** | **(95% CI)** | **P value** | **Patients/No of deaths** | **HR** | **(95% CI)** | **P value** |
| Tumour centre CD8 |  |  |  |  |  |  |  |  |  |  |  | **Tumour centre CD8** |  |  |  |
| High | 73/22 | 128/41 | 1.16 | 0.69-1.95 | 0.56 | 1.48 | 0.84-2.59 | 0.165 | 1.61 | 0.91-2.86 | 0.10 | 215/51 | 1.34 | 0.73-2.47 | 0.34 |
| Low | 67/27 | 119/44 | 1.07 | 0.66-1.74 | 0.76 | 1.02 | 0.60-1.73 | 0.923 | 1.02 | 0.60-1.73 | 0.94 | 195/59 | 1.09 | 0.61-1.94 | 0.77 |
| p for interactions |  |  |  |  | 0.56 |  |  |  |  |  |  |  |  |  | 0.96 |
| Invasive edge CD8 |  |  |  |  |  |  |  |  |  |  |  | **Invasive edge CD8** |  |  |  |
| High | 69/19 | 126/35 | 1.13 | 0.64-1.97 | 0.67 | 1.36 | 0.75-2.48 | 0.301 | 1.38 | 0.75-2.52 | 0.29 | 203/43 | 1.52 | 0.78-2.98 | 0.22 |
| Low | 64/28 | 107/45 | 1.05 | 0.65-1.69 | 0.82 | 1.04 | 0.63-1.73 | 0.857 | 1.02 | 0.60-1.70 | 0.94 | 182/60 | 0.87 | 0.50-1.52 | 0.64 |
| p for interactions |  |  |  |  | 0.67 |  |  |  |  |  |  |  |  |  | 0.59 |
| Overall survival | | | | | | | | | | | | | | | |
| Tumour centre CD8 |  |  |  |  |  |  |  |  |  |  |  | **Tumour centre CD8** |  |  |  |
| High | 88/37 | 143/56 | 0.94 | 0.62-1.42 | 0.77 | 1.13 | 0.72-1.77 | 0.58 | 1.20 | 0.76-1.90 | 0.41 | 215/77 | 1.13 | 0.69-1.84 | 0.61 |
| Low | 86/46 | 130/55 | 0.79 | 0.53-1.17 | 0.25 | 0.80 | 0.52-1.23 | 0.31 | 0.79 | 0.51-1.22 | 0.30 | 195/80 | 0.89 | 0.55-1.46 | 0.67 |
| p for interactions |  |  |  |  | 0.29 |  |  |  |  |  |  |  |  |  | 0.13 |
| Invasive edge CD8 |  |  |  |  |  |  |  |  |  |  |  | **Invasive edge CD8** |  |  |  |
| High | 81/31 | 136/45 | 0.90 | 0.57-1.42 | 0.66 | 1.06 | 0.65-1.73 | 0.80 | 1.11 | 0.68-1.81 | 0.67 | 203/62 | 1.21 | 0.70-2.08 | 0.49 |
| Low | 82/46 | 122/60 | 0.85 | 0.58-1.25 | 0.42 | 0.87 | 0.58-1.32 | 0.53 | 0.87 | 0.57-1.32 | 0.52 | 182/84 | 0.81 | 0.51-1.29 | 0.39 |
| p for interactions |  |  |  |  | 0.35 |  |  |  |  |  |  |  |  |  | 0.75 |

- Excluded patients with previous history of CRC, or hereditary linked CRC from the analysis.

- Unknown tumour centre and maximum immune biomarker status removed.

*Adjusted; sex, chemotherapy, age, stage, ECOG status and tumour grade differentiation.

**Adjusted+MSI; sex, chemotherapy, age, stage, ECOG status, tumour grade differentiation and MSI.

Supplementary Table 9 Alcohol consumption and colorectal cancer-specific survival and overall survival by FOXP3 status.

| CRC-specific survival | | | | | | | | | | | | | | | |
| --- | --- | --- | --- | --- | --- | --- | --- | --- | --- | --- | --- | --- | --- | --- | --- |
|  | **Never**  **alcohol** | **Ever**  **alcohol** | **Unadjusted** | | | **Adjusted*** | | | **Adjusted+MSI **** | | | **Adjusted sensitivity analysis *** | | | |
|  | **Patients/ No. of deaths** | | **HR** | **(95% CI)** | **P value** | **HR** | **(95% CI)** | **P value** | **HR** | **(95% CI)** | **P value** | **Patients/No of deaths** | **HR** | **(95% CI)** | **P value** |
| Tumour centre FOXP3 | |  |  |  |  |  |  |  |  |  |  | **Tumour centre FOXP3** |  |  |  |
| High | 76/23 | 118/39 | 1.18 | 0.70-1.99 | 0.51 | 1.17 | 0.68-2.02 | 0.56 | 1.16 | 0.67-2.03 | 0.58 | 205/50 | 1.08 | 0.59-1.97 | 0.79 |
| Low | 64/26 | 126/45 | 1.02 | 0.63-1.66 | 0.92 | 1.18 | 0.69-2.03 | 0.53 | 1.16 | 0.68-2.00 | 0.57 | 201/59 | 1.24 | 0.69-2.24 | 0.47 |
| p for interactions | |  |  |  | 0.60 |  |  |  |  |  |  |  |  |  | 0.87 |
| Invasive edge FOXP3 | |  |  |  |  |  |  |  |  |  |  | **Invasive edge FOXP3** |  |  |  |
| High | 80/22 | 92/30 | 1.27 | 0.73-2.20 | 0.39 | 1.48 | 0.82-2.66 | 0.19 | 1.59 | 0.87-2.91 | 0.13 | 184/43 | 1.49 | 0.78-2.85 | 0.23 |
| Low | 48/23 | 119/46 | 0.86 | 0.52-1.42 | 0.57 | 1.18 | 0.66-2.09 | 0.57 | 1.15 | 0.65-2.05 | 0.61 | 175/56 | 1.15 | 0.61-2.15 | 0.66 |
| p for interactions |  |  |  |  | 0.85 |  |  |  |  |  |  |  |  |  | 0.39 |
| Overall survival | | | | | | | | | | | | | | | |
| Tumour centre FOXP3 | |  |  |  |  |  |  |  |  |  |  | **Tumour centre FOXP3** |  |  |  |
| High | 88/35 | 133/54 | 1.08 | 0.70-1.66 | 0.71 | 1.12 | 0.71-1.77 | 0.61 | 1.14 | 0.72-1.81 | 0.56 | 205/73 | 1.04 | 0.63-1.71 | 0.87 |
| Low | 84/46 | 136/55 | 0.70 | 0.49-1.04 | 0.09 | 0.77 | 0.50-1.18 | 0.23 | 0.75 | 0.48-1.15 | 0.19 | 201/82 | 0.86 | 0.54-1.39 | 0.57 |
| p for interactions | |  |  |  | 0.33 |  |  |  |  |  |  |  |  |  | 0.46 |
| Invasive edge FOXP3 | |  |  |  |  |  |  |  |  |  |  | **Invasive edge FOXP3** |  |  |  |
| High | 93/35 | 103/41 | 1.10 | 0.70-1.73 | 0.66 | 1.45 | 0.89-2.38 | 0.13 | 1.55 | 0.94-2.56 | 0.08 | 184/64 | 1.60 | 0.93-2.75 | 0.09 |
| Low | 63/38 | 132/59 | 0.67 | 0.44-1.01 | 0.06 | 0.87 | 0.55-1.39 | 0.57 | 0.87 | 0.54-1.38 | 0.57 | 175/77 | 0.92 | 0.55-1.55 | 0.78 |
| p for interactions | |  |  |  | 0.27 |  |  |  |  |  |  |  |  |  | 0.16 |

- Excluded patients with previous history of CRC, or hereditary linked CRC from the analysis.

- Unknown tumour centre and maximum immune biomarker status removed.

*Adjusted; sex, chemotherapy, age, stage, ECOG status and tumour grade differentiation.

**Adjusted+MSI; sex, chemotherapy, age, stage, ECOG status, tumour grade differentiation and MSI.

*Adjusted sensitivity analysis; sex, chemotherapy, age, stage, ECOG status and tumour grade differentiation.
